# Comparison of immunohistochemical and qPCR methods from granulomatous dermatitis lesions for detection of leishmania in dogs living in endemic areas: a preliminary study

**DOI:** 10.1186/s13071-022-05218-6

**Published:** 2022-03-24

**Authors:** Ilaria Porcellato, Giulia Morganti, Maria Teresa Antognoni, Katarzyna Małgorzata Walczak, Stefano De Arcangeli, Tommaso Furlanello, Cristina Bianca Quattrone, Fabrizia Veronesi, Chiara Brachelente

**Affiliations:** 1 Department of Veterinary Medicine, University of Perugia, Via San Costanzo 4, 06126 Perugia, Italy; 2 Faculty of Veterinary Medicine and Animal Science, Poznań University of Life Sciences, Wolynska 33, 60-637, Poznań, Poland; 3 San Marco Veterinary Clinic and Laboratory, Via dell’Industria 3, 35030 Padova, Italy; 4 Consulting Statistician, Via Salazar 2, 89123, Reggio Calabria, Italy

**Keywords:** Immunohistochemistry, Dog, Granulomatous dermatitis, *Leishmania*, qPCR

## Abstract

**Background:**

In canine leishmaniosis (CanL) endemic areas, pathologists often receive skin biopsies for testing with histopathologic findings suggestive—but not conclusive for a definitive diagnosis—of CanL lesions. I the absence of data on the infective status of animals, the diagnosis can therefore be challenging. The aim of this retrospective study was to evaluate the ability of immunohistochemistry (IHC) and quantitative PCR (qPCR) methods to detect *Leishmania* infection in skin biopsies with a histopathologic diagnosis of lymphoplasmacytic/histiocytic and/or granulomatous dermatitis and to correlate the pattern, depth and severity of the histopathologic lesions with the parasite load detected by qPCR and IHC.

**Methods:**

Thirty formalin-fixed, paraffin-embedded skin samples were evaluated by hematoxylin–eosin (H&E) staining, IHC, conventional PCR (cPCR) and qPCR. The severity, pattern and depth of the dermal inflammation and parasite load were graded.

**Results:**

*Leishmania* was detected by H&E staining in 8/30 sections (26.66%) and by IHC in 14/30 samples (46.66%). Parasite DNA was detected in 14/30 samples (46.66%) by cPCR and in 21/30 samples (70%) by qPCR, with an extremely variable parasite load (1.32–62.700 copies). The level of agreement was fair between H&E staining and cPCR (κ = 0.32), and moderate between H&E staining and IHC (κ = 0.58). The level of agreement between IHC and cPCR was good (κ = 0.65); between IHC and qPCR, moderate (κ = 0.41); and between cPCR and qPCR, fair (κ = 0.28). A significant association was found between the severity of dermal inflammation and the parasitic skin load by IHC, although with weak linear correlation.

**Conclusions:**

Our study underlines the difficulty of obtaining a definitive diagnosis of CanL cutaneous lesions, even with the most accurate diagnostic tests currently available. Based on our results, no single test is suitable on its own for the diagnosis of cutaneous lesions caused by *Leishmania*. However, in the presence of a moderate/severe lymphoplasmacytic/histiocytic and/or granulomatous dermatitis, we suggest performing IHC, as in our study this technique proved to be the method with the highest discriminatory power to estimate the role of the parasite in skin lesions. In mild lesions, IHC loses its discriminatory power and should be effectively combined with techniques such as qPCR.

**Graphical Abstract:**

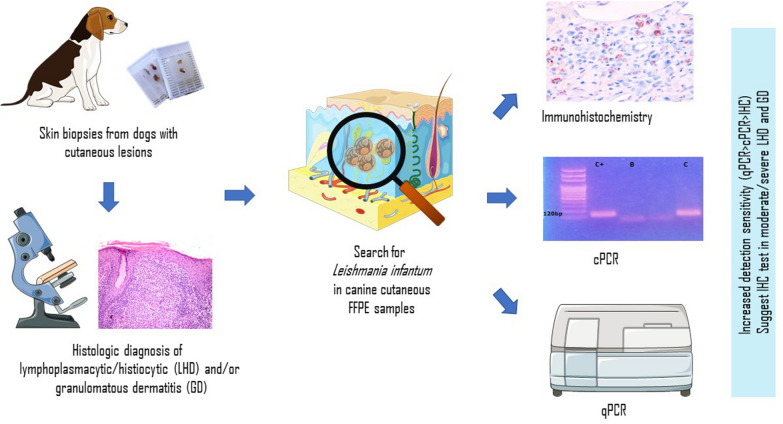

## Background

Most dogs living in endemic areas of canine leishmaniosis (CanL) are constantly exposed to being bitten by sand flies and thus to *Leishmania* infection. However, only relatively few animals develop the disease, depending on whether their immune system is adequate to control parasite multiplication and tissue invasion.

The skin is the organ where the first interaction between the parasite and the canine immune system occurs [1]. Skin lesions are thus frequently the most common finding (67–89% of cases) in dogs infected with *Leishmania* upon physical examination. Such lesions can appear in isolation or in various combinations with other clinicopathologic abnormalities [2, 3].

Cutaneous manifestations of CanL are extremely pleomorphic and may be grouped into typical and atypical forms [4, 5]. In the typical forms (e.g. exfoliative dermatitis, ulcerative dermatitis of pressure points, etc.), the clinical features are highly suggestive of CanL and, according to the guidelines suggested by the Canine Leishmaniasis Working Group (CLWG) [6], veterinarians should follow the diagnostic algorithm, including indirect tests [i.e. indirect fluorescence antibody test (IFAT), enzyme-linked immunosorbent assay (ELISA) and direct agglutination test (DAT)] and direct tests aimed at the direct visualization of the parasite or amplification of the parasitic DNA [7]. Skin biopsies and histopathologic examination can be carried out in these cases to confirm the clinical suspicion of CanL. However, in endemic areas, several atypical forms can also occur, such that the cutaneous lesions are not highly suspicious of CanL and indeed are similar to those of other skin diseases with a completely different pathogenesis (i.e. pemphigus foliaceus, etc.). In these cases, the referring veterinarians may be completely unaware of the possibility that *Leishmania* may cause the observed lesions, resulting in the possibility that the cutaneous biopsies can be sent to the pathologist without mention of a specific suspect cause and with no preliminary (direct and indirect) tests performed.

Histopathologic findings in skin biopsies are not specific for CanL and consist of varying degrees of perivascular to interstitial, and focal to nodular to diffuse inflammatory infiltrate in the dermis, with various combinations of macrophages, plasma cells, lymphocytes, mast cells and isolated neutrophils [8–10]. These patterns may be shared by other infectious and non-infectious/immune-mediated diseases (i.e. sterile granuloma and pyogranuloma syndrome, granulomatous forms of sebaceous adenitis, etc.) [11]. Therefore, the pathologist diagnosing a lymphoplasmacytic/histiocytic dermatitis (LHD) and/or granulomatous dermatitis (GD) in skin biopsies from dogs with cutaneous lesions compatible with CanL has to rule out *Leishmania* in the list of differential diagnoses.

Upon histopathology of the skin biopsies, a definitive diagnosis of *Leishmania* dermatitis should be based on the demonstration of the parasite in the inflammatory foci. However, examination of histological sections stained with hematoxylin–eosin (H&E) alone is frequently inconclusive due to (i) the possibility of there being an insufficient number of amastigotes of *Leishmania*; and (ii) the variable rate at which amastigotes can be seen in cases (14–80%) [5]. The development of the immunohistochemistry (IHC) technique has substantially improved diagnosis based on histopathology owing to the former’s higher sensitivity and specificity (18.2–100% of cases) [12, 13], together with its ability to correlate intralesional *Leishmania* amastigotes with the pathogenesis of lesions [14]. However, doubtful cases still emerge when the inflammatory infiltrate is compatible with *Leishmania* but neither H&E staining nor IHC are able to detect intralesional parasites. In practice, molecular methods (i.e. PCR) associated with histopathology are thus adopted as alternative or additional methods for diagnosing CanL cutaneous forms [15].

 PCR on skin biopsies seems to be superior to other techniques in terms of sensitivity and specificity to detect *Leishmania* [2, 10, 15, 16]. However, the molecular identification of parasite DNA simply confirms the infection and that *Leishmania* is present in the skin; it does not necessarily indicate that the cutaneous lesions are the result of CanL [14].

Quantitative PCR (qPCR) overcomes some of the drawbacks of conventional PCR (cPCR) techniques, such as reducing the risk of contamination, decreasing the assay time and improving the detection limit [17–19]. It also gives a better correlation with the pathogenesis of the lesions alone or in association with IHC.

The aim of the retrospective study reported here was to evaluate the ability of IHC and qPCR methods to detect *Leishmania* infection in formalin-fixed and paraffin-embedded (FFPE) skin sections from dogs living in an endemic area for CanL and having a histopathologic diagnosis of LHD and/or GD without any previous suspicion of CanL (no suspicion or record of any preliminary tests in the history sheet). A detailed histopathologic examination was also conducted to demonstrate a possible correlation between the severity, pattern and depth of dermal inflammation and the parasite load as determined by qPCR and IHC. Identifying a pattern, depth and severity of dermal inflammation significantly associated with the parasite, and possibly the parasitic burden, would be useful to guide histopathologic diagnosis and choice of complementary tests to be performed or suggested on FFPE samples.

## Methods

### Skin sampling collection

The database repository of the Veterinary Pathology Service of the Veterinary Teaching Hospital (OVUD) of the Department of Veterinary Medicine of Perugia (central Italy) was searched to identify histopathology records of skin biopsies from dogs living in CanL endemic areas, namely those dogs recently estimated to be at medium–high risk (prevalence rates: 10–20%) for CanL [20].

The skin FFPE blocks of the dogs were included in the study if: (i) the histopathology previously performed by a board-certified (CB) veterinary pathologist confirmed features consistent with LHD and/or GD compatible with *Leishmania* infection; and (ii) an adequate amount of FFPE tissue (> 0.5 cm^2^) was stored in the biorepository at the time of the investigation. The present study, therefore, included 30 dogs whose samples has been collected between 2005 and 2020.

### Histopathology and immunohistochemistry

Slides containing 4-µm-thick sections were prepared from the skin FFPE blocks and stained with H&E for histopathologic examination. H&E-stained slides were checked for lesions in the dermis as follows: inflammation (presence/absence); severity (mild, moderate, severe); pattern (perivascular, interstitial, band-like, nodular, diffuse); depth (superficial, mid dermis, deep); and relative percentage of inflammatory cells (macrophages, lymphocytes, plasma cells, neutrophils, multinucleated giant cells, eosinophils and mast cells). H&E staining was used to characterize amastigote forms of *Leishmania* according to their size, shape and location inside macrophages, and to estimate the parasite load. The number of microscopic fields that were positive for *Leishmania* amastigotes was counted, and the percentage of positive fields was calculated based on the average number of positive fields per skin sample. The grading system used for H&E-stained samples was: (i) negative (−, no amastigotes found); (ii) suspect [+/−, < 5% positive microscopic fields, at 400× magnification, field number (FN) = 22 mm], or samples where the detection of parasites was inconclusive or difficult and where differentiation from phagocytosed cell debris was necessary); (iii) positive [+, with mild parasite load (5–25% positive microscopic fields); ++, moderate parasite load (25–50% positive microscopic fields); +++, intense parasite load (> 50% positive microscopic fields)]. A minimum of 10 high-power fields at 400× magnification were evaluated.

For IHC, a protocol described by Tafuri et al. [13] was used, with slight modifications. Briefly, immunohistochemical labeling was performed on 4-μm-thick serial sections mounted on poly-L-lysine-coated slides. After deparaffination in xylene and rehydration in graded alcohols, antigen retrieval was performed in a microwave by immersion of slides in a pre-heated citrate solution (pH 6.0). The slides were then washed with Tris-buffered saline (TBS) buffer and incubated in 3% H_2_O_2_ for 10 min. After protein blocking (ab93677; Abcam, Cambridge, UK), the slides were incubated overnight in a humidified chamber with the serum of a dog naturally infected with *Leishmania infantum* (titre 1:320), at a 1:2000 dilution, which was applied as the primary antibody. As a secondary antibody, a horseradish peroxidase-conjugated goat polyclonal anti-dog antibody (ab112835; Abcam) was applied at 1:200 dilution and incubated for 1 h at room temperature. After the secondary antibody, immunolabeling was revealed with DAB (3,3′-diaminobenzidine; ab64238; Abcam), and Mayer’s hematoxylin was applied as a counterstain. A known positive skin sample from a sick dog due to CanL was used as a positive control. Negative controls were incubated with TBS, omitting the primary antibody. In addition, skin sections collected at necropsy from PCR-negative dogs from a non-endemic country were used as negative controls. The skin tissue parasite loads were semi-quantitatively analyzed by the presence of immunolabeled amastigote forms of *Leishmania* associated with the chronic inflammatory reaction in the dermis at 400× magnification; the grading system was the same as that described for the morphological (H&E) examination.

### Molecular methods

Five 5-mm-thick sections of each FFPE skin tissue block were cut and handled with a new disposable razor blade and new gloves to prevent cross-contamination with *Leishmania* DNA. After each block was cut, the microtome blade, tweezers and entire cutting area were carefully cleaned with a 0.1 M solution of sodium hypochlorite to break down any potential contamination.

The sections were deparaffinized at room temperature by two consecutive immersion washes, 30 min each wash, in 1 ml of xylene and then rinsed twice, 5 min each rinse, with 1 ml of 100% ethanol. The samples were centrifuged at 10,000 *g* for 5 min, and the liquid was decanted between each change. Total genomic DNA was extracted using the ExgeneTM Clinic SV Mini Kit (GeneAll, Seoul, Korea), according to the manufacturer’s protocol. The extracted DNA was used in a cPCR protocol to amplify a final 120-bp fragment of conserved region of the *Leishmania* kinetoplastic (k) DNA minicircle, according to Francino et al. [21]. The *Leishmania*-specific primers used were NP13A (forward: 5′- AACTTTTCTGGTCCTCCGGG -3′) and NP13B (reverse: 5′- CCCCCAGTTTCCCGCCC -3′). Reaction mixtures were prepared in a total reaction volume of 50 μl that contained 25.0 μl of EconoTaq PLUS GREEN 2× Master mix (Lucigen Corporation, Middleton, WI, USA), 1 μM of sense primer, 1 μM of reverse primer and 1 μl of extracted DNA, ranging from 50 to 100 ng for each reaction. The conditions for the cPCR amplification were: initial denaturation at 94 °C for 5 min; denaturation at 94 °C for 30 s, annealing at 58 °C for 30 s and extension at 72 °C for 1 min, for 50 cycles; and a final extension at 72 °C for 5 min.

The reaction was carried out in a StepOnePlus™ instrument (Applied Biosystems, Thermo Fischer Scientific, Foster City, CA, USA). Each reaction included a negative (sterile water) and a positive control (DNA extracted from *L. infantum*-cultured promastigotes).

Samples (each sample: 15 μl) of the amplification products were electrophoresed in a 1.2% agar gel for 30 min at 100 V in TBE buffer (89 mM Tris borate, 2.0 mM EDTA, pH 8.3) with 5 ul of EuroSafe Nucleic Acid Stain (EuroClone S.p.A., Pero, Italy) and 10 ul of SharpMassTM 100 ready-to load DNA ladder (100 bp) (EuroClone S.p.A.) to determine the PCR fragment size. The gel was visualized under UV transillumination.

The amplicons obtained from cPCR were directly sequenced in both directions using a 16-capillary ABI PRISM 3130 × l Genetic Analyzer, assembled and edited with SeqScape software v 2.5 (all Applied Biosystems). The assembled sequences were compared to *Leishmania* spp. sequences available in GenBank using the Basic Local Alignment Search Tool (BLAST; https://blast.ncbi.nlm.nih.gov/; accessed on 05 Feb 2022).

Detection and quantification of *L. infantum* DNA was performed using a real-time PCR commercial kit (TIB Molbiol, Genova, Italy), as described by Solano-Gallego et al. [22]. The kit was based on a couple of primers and a fluorescent resonance energy transfer (FRET) probe specific for *L. infantum* kinetoplast DNA minicircles. Real-time PCR was carried out using LightCycler FastStart DNA MasterPLUS Hybridization Probes (Roche, Mannheim, Germany) in a LightCycler II instrument (Roche); the composition of the reaction mix and thermal cycling conditions were according to the manufacturer’s protocol. A no-template control (water) and a negative control were included in the qPCR run in order to exclude the risk of contamination. Parasite load was quantified using the absolute quantification method. Serial tenfold dilutions of recombinant plasmid containing the target DNA with a known copy number (ranging from 1 × 108 to 1 × 100 copies/μl) were used to generate the qPCR standard curve. Results were expressed as target DNA copies per microliter (copies/μl) of extract.

A grading system based on the amount of target DNA copies per microliter of extract was used. Samples were arbitrarily classified as negative (−, no amplification), low (+, 1–100 copies/μl), moderate (++, 101–10,000 copies/μl) or intense (+++, > 10,000 copies/μl).

### Statistical analysis

The frequencies of positive results obtained from all the skin samples through the diagnostic tests (e.g. H&E staining, IHC, cPCR and qPCR) with 95% confidence intervals (CI) were calculated and compared using the Chi-square test (*χ*2) test. An accepted level of significance was set at *p* < 0.05. In addition, the degree of agreement between the evaluated tests was determined by the Kappa coefficient (κ) value and interpreted as follows: κ = 0.01–0.20, scant agreement; κ = 0.21–0.4, fair agreement; κ = 0.41–0.60, moderate agreement; κ = 0.61–0.80, good agreement; and κ ≥ 0.80, almost total agreement.

A preliminary statistical descriptive analysis of the dependent variables was performed taking into consideration the severity, the depth and the pattern of dermal inflammation. The position indices (median and mode) were calculated in order to find the “central trend” of the variables, as well as to determine the value corresponding to the maximum observed absolute frequency. The associations between the histologic variables and grading of the parasitic load detected by H&E staining, IHC and qPCR were assessed using the *χ*2 test. To verify any correlations between variables, we used the Pearson test (Gaussian distribution data) and the Spearman test (non-normal data). Associations between histological variables and the presence of the parasite were assessed with the Mann–Whitney U-test. The calculations were carried out using the SPSS computer program for epidemiologist V.11.30 (IBM Corp., Armonk, NY, USA).

## Results

The overall results for the positivity rates detected by H&E staining, IHC, cPCR and qPCR techniques are shown in Table 1.

**Table 1:** *Leishmania* positivity rates as detected by H&E staining, IHC, cPCR and qPCR

Diagnostic methods	*N* (%)	95% CI
H&E staining	8 (26.66%)	12.3–45.9%
IHC	14 (46.66%)	28.3–65.7%
cPCR	14 (46.66%)	28.3–65.7%
qPCR	21 (70%)	50.6–85.3%

CI, Confidence interval

The qPCR technique had the highest rate for detecting *Leishmania*, followed, in order of decreasing detection rate, by IHC and cPCR and H&E. The results of the severity, pattern and depth of the dermal inflammatory infiltrates as well as the parasite load graded using histologic, IHC and biomolecular methods are shown in Table 2.

**Table 2:** Inflammatory infiltrate (graded histologically) and parasite load (graded by histologic, IHC and biomolecular techniques) in the skin samples analyzed

Skin sample ID	Severity of dermal inflammation	Pattern of dermal inflammation	Depth of dermal inflammation	cPCR	Grading of parasite load		
					H&E staining^a^	IHC^a^	qPCR^a^
1	Moderate	Interstitial	Deep	Positive	++	++	+++
2	Severe	Nodular	Deep	Positive	+++	+++	+++
3	Severe	Interstitial	Mid dermis	Positive	++	+++	+++
4	Moderate	Band-like	Mid dermis	Positive	+++	+++	++
5	Severe	Nodular	Deep	Positive	++	+	+++
6	Moderate	Interstitial	Mid dermis	Positive	+	+	+++
7	Severe	Diffuse	Deep	Positive	±	+	++
8	Severe	Nodular	Deep	Positive	±	+	++
9	Moderate	Perivascular	Mid dermis	Positive	+/−	++	+++
10	Severe	Diffuse	Mid dermis	Positive	−	+	+++
11	Moderate	Interstitial	Mid dermis	Positive	−	+/−	+++
12	Moderate	Nodular	Mid dermis	Positive	−	−	+
13	Severe	Diffuse	Deep	Positive	−	+	−
14	Mild	Perivascular	Superficial	Negative	+++	+++	+++
15	Moderate	Interstitial	Mid dermis	Negative	+++	++	+
16	Severe	Nodular	Deep	Negative	−	+	++
17	Moderate	Band-like	Deep	Negative	+/−	−	+ +
18	Moderate	Band-like	Superficial	Negative	−	−	+
19	Mild	Perivascular	Superficial	Negative	−	−	+
20	Mild	Perivascular	Mid dermis	Negative	−	−	+
21	Mild	Interstitial	Mid dermis	Negative	−	−	+
22	Moderate	Interstitial	Mid dermis	Negative	−	−	+
23	Severe	Nodular	Deep	Positive	−	−	−
24	Severe	Nodular	Deep	Negative	+/−	−	−
25	Severe	Diffuse	Deep	Negative	−	−	−
26	Mild	Perivascular	Superficial	Negative	−	−	−
27	Moderate	Noduslar	Deep	Negative	−	−	−
28	Moderate	n.a.	n.a	Negative	−	−	−
29	Mild	Perivascular	Superficial	Negative	−	−	−
30	Moderate	Perivascular	Mid dermis	Negative	−	−	−

n.a., Not applicable

^a^See Methods section for a description of the grading system applied

**Fig. 1:**
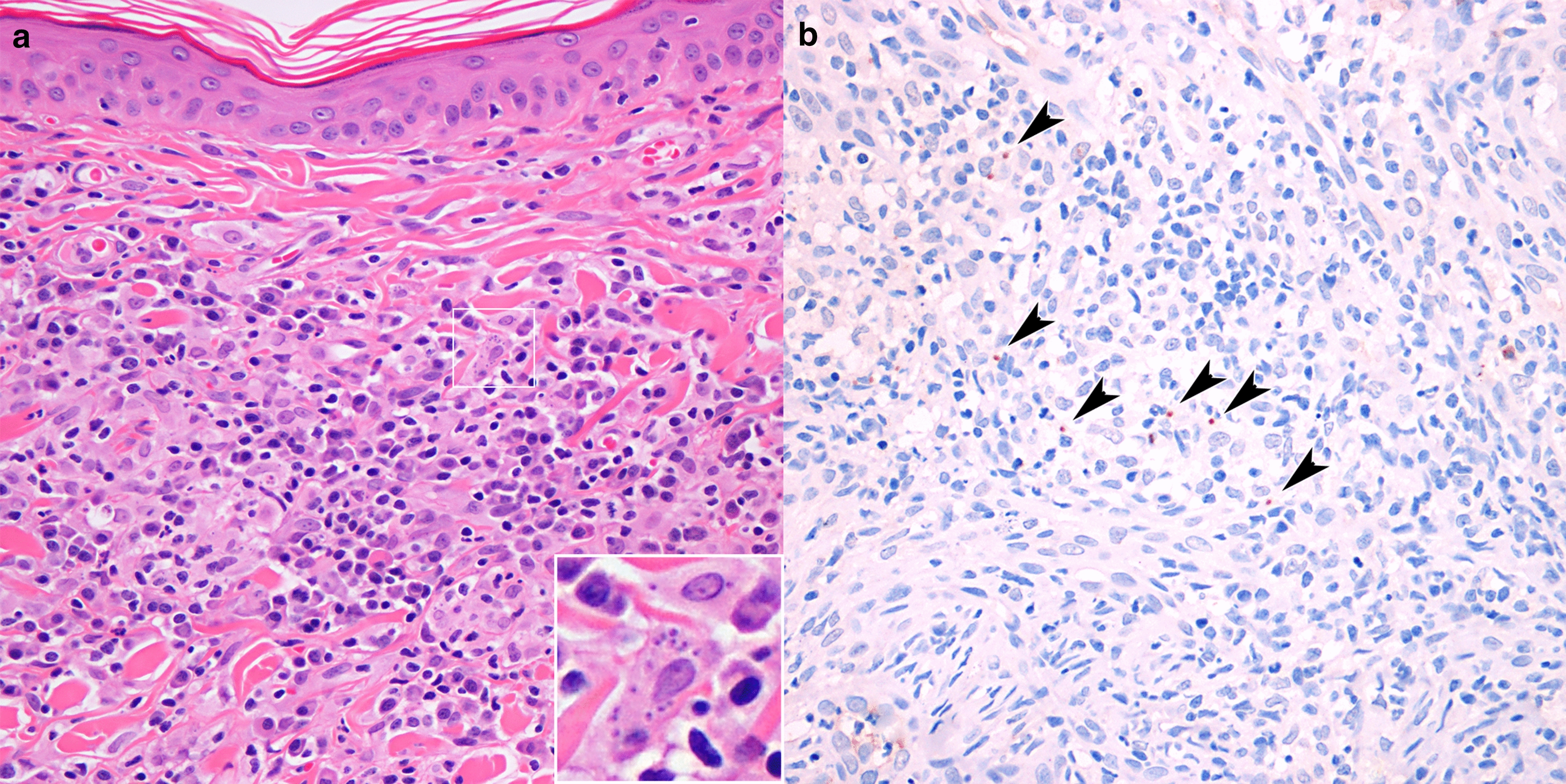
Morphological and immunohistochemical features of *Leishmania* dermatites. **a** An interstitial, multifocal image of coalescing, lymphoplasmacytioc amd histiocyitc dermatites in the superficial dermis. Insert: Amastigotes of* Leishmania* can be seen in the cytoplasm of macrophages (H&E, 40×; ID number: 3). **b** Immunohistochemistry showing positivity for anti-*Leishmania* antibody in the cytoplasm of macrophages, as indicated by the arrowheads (IHC, 40×; ID number: 6)

Amastigote forms of *Leishmania* in H&E-stained sections were visualized in 8/30 samples (positivity rate: 26.66%); five skin samples (16.66%) were considered suspicious (+/−) for *Leishmania* but the results were inconclusive. No amastigotes were detected in the remaining 17 skin samples (56.68%). The parasite load detected by H&E staining was mild (+) in 3.33% (1/30) of skin samples, moderate (++) in 10% (3/30) (Fig. 1a) and intense (+++) in 13.33% (4/30). The results of the IHC revealed that 14/30 (46.66%) of the skin samples were positive for *Leishmania*, while the findings for one (3.33%) skin sample was considered to be inconclusive for amastigote. In 15 skin samples (50.00%), no amastigotes were detected. The parasite load within lesions was considered to be mild (+) in seven cases (23.33%) (Fig. 1b), moderate (++) in three cases (10%) and intense (+++) in four cases (13.33%).

Parasite DNA was detected in the analyzed tissue in 14/30 samples (46.66%) by cPCR, and sequencing of amplicons confirmed that *L. infantum* was the species isolated; in comparison, 21/30 skin samples (70%) tested positive by qPCR (Table 2). The parasite load within lesions detected by qPCR was extremely variable, ranging from 1.32 to 62.700 copies. Depending on the grading system adopted, there was a low (+) parasitic load in seven samples (23.33%), a moderate (++) parasitic load in five samples (16.66%) and an intense parasitic load (+++)(30%) in nine tissue samples.

Of the 30 skin samples, 23 tested positive for *Leishmania*, as demonstrated by the results of the DNA amplification of the parasite and/or the presence of visible amastigotes by histologic or immunohistochemical assay. Of these 23 *Leishmania*-positive skin samples, six with predominantly moderate or severe parasitic loads were positive in all four tests, ten were positive in at least two diagnostic techniques and seven were only positive for one of the molecular techniques (cPCR or qPCR). The remaining seven samples were totally negative for all the tests applied (Table 2). Based on these results, we identified two groups: Group A, which consisted of samples that tested positive for the diagnosis of *Leishmania* dermatitis (*n* = 16 cases), with morphologic evidence of the parasite in the skin lesions and positive molecular tests supporting the morphologic results; and Group B, which consisted of skin samples that tested negative for the diagnosis of *Leishmania* dermatitis (*n* = 14 cases) and for which no parasite could be demonstrated by any of the tested techniques, and samples that, despite testing positive for parasite DNA amplification, the actual presence of the parasite in the skin lesions could not be demonstrated.

Regarding the main histopathologic findings observed in the skin biopsies, the most common dermal change found was a moderate (13/30, 43.3%), nodular (8/30, 26.6%) inflammation located in the mid or deep dermis (both 12/30, 40% each). However, considering Group A and Group B separately, there was a statistically significant difference in the degree of inflammation between the positive and negative samples (*p* < 0.037). In skin samples in Group A, the inflammatory infiltrate was predominantly moderate (7/16, 43.7%) or severe (8/16, 50%), whereas in those of Group B, dermal inflammation was predominantly mild (5/14, 35.7%) or moderate (6/14, 42.9%). Although not statistically significant, the positive group more often had a nodular (5/16, 31.3%), interstitial (5/16, 31.3%) or diffuse (3/16, 18.7%) distribution of inflammation, with a depth in the mid (8/16, 50%) or deep (7/16, 43.8%) dermis. On the other hand, the negative group more often had a perivascular (5/14, 35.7%) or nodular (3/14, 21.5%) distribution of inflammation, localized in the superficial (4/14, 28.6%) or mid (4/14, 28.6%) dermis. Overall, the severity of inflammation correlated with the depth of the inflammatory infiltrate (ρ = 0.741, *p* < 0.001).

Table 3 shows a comparison of the results obtained with the H&E staining, IHC and biomolecular techniques (i.e. cPCR and qPCR) and their relative agreement.

**Table 3:** Cross-comparison and agreement of paired tests (H&E, IHC, cPCR and qPCR)

Diagnostic methods	Diagnostic method *N* (%)	*χ*^2^ (*p*)/Overall agreement (k)			
		H&E staining [a]	IHC [b]	cPCR [c]	qPCR [d]
H&E staining [a]	8 (26.66%)	ne/ne	–	–	–
IHC [b]	14 (46.66%)	[a] vs [b]: 2.58(0.1)/ 0.8 (0.58)	ne/ne		–
cPCR [c]	14 (46.66%)	[a] vs [c]: 2.58 (0.1)/ 0.66 (0.32)	[b] vs [c]: 0 (1)/ 0.8(0.65)	ne/ne	–
qPCR [d]	21 (70%)	[a] vs [d]:11.28 (< 0.001*)/ 0.56 (0.27)	[b] vs [d]: 1.76 (0.18)/ 0.7 (0.41)	[c] vs [d]: 1.76 (0.18) / 0.63 (0.28)	sne/ne

*n* = 30 skin samples

κ, Kappa coefficient, ;  ne, not estimated

*Statistical difference between methods for the same sample

No statistical difference in the rates of positivity was observed between the H&E staining, cPCR and IHC techniques (*p* = 0.1), although a lower number of positive samples was detected with H&E staining than with the cPCR and IHC methods [8 vs 14 [for cPCR and IHC, respectively]). The level of agreement between H&E and cPCR was fair (66%, κ = 0.32), and that between H&E and IHC was moderate (80%, κ = 0.58). All the H&E-positive tissue samples were also positive by IHC. Of the five skin samples found to be suspicious but inconclusive by H&E staining, three were confirmed to be positive by cPCR and IHC, with a mild to moderate parasitic load. The agreement between IHC and cPCR was good (κ = 0.65), except in five samples where cPCR was positive and IHC was negative (2/5), or the other way round (3/5). The rate of positivity detected by IHC and cPCR was lower than that obtained by qPCR (14 vs 21), but the difference was not significant (*p* = 0.18). Moderate agreement (70%, κ = 0.41) was found between IHC and qPCR and fair agreement (63%, κ = 0.28) was found between cPCR and qPCR. Of the 14 positive IHC samples, 13 were also positive according to qPCR; however, qPCR detected six additional positive samples compared to IHC.

The rates of positivity detected by H&E staining and qPCR were statistically different (*p* < 0.001), with a higher number of positives (21 vs 8) detected by qPCR and a a fair degree of agreement (56%, κ = 0.27). All of the positive samples by H&E staining were also positive by qPCR. Four of the five skin samples found to be suspicious but inconclusive by H&E staining were confirmed to be positive by qPCR, with mainly a moderate parasitic load.

No association between any of the variables tested was found (*p* > 0.05), with the exception of a significant association between the severity of dermal inflammatory infiltrate and parasitic skin load detected by IHC (*p* = 0.029) (Table 4). However, a weak linear correlation was detected (*r* = 0.26, *p* = 0.099).

**Table 4:** Distribution of the parasitic load detected using H&E staining, IHC and qPCR, and the association with severity of dermal inflammation

Severity of dermal inflammation (*N*)	Parasite load													
	H&E staining^a^					IHC^a^					qPCR^a^			
	Negative (−)	Suspect (+/–)	Mild (+)	Moderate (++)	Intense (+++)	Negative (−)	Suspect (+/–)	Mild (+)	Moderate (++)	Intense (+++)	Negative (−)	Low (+)	Moderate (++)	Intense (+++)
Mild (+) (6)	5	0	0	0	1	5	0	0	0	1	2	3	0	1
Moderate (++) (13)	7	2	1	1	2	7	1	1	3	1	3	4	2	4
Severe (+++) (11)	5	3	0	2	1	3	0	6	0	2	4	0	3	4
*χ*2 (*p*)	5.7 (0.77)					15.64 (0.029)*					7.34 (0.29)			
*r* (*p*)	0.18 (0.33)					0.26 (0.099)					0.13 (0.41)			

Numbers in table are the number of samples, unless indicated otherwise

*Statistical difference

^a^See Methods section for a description of the grading system applied

## Discussion

In the present study we evaluated the ability of different techniques and methods, such as IHC and qPCR, to detect *Leishmania* infection in skin samples that have histologic findings compatible or suggestive of CanL. These techniques are often used, or at least have been proposed to be used in a histopathologic diagnostic setting due to the clinical-anamnestic data frequently being incomplete. Demonstrating the presence of *Leishmania* within the skin lesion is often essential to a diagnosis of CanL due to the histopathologic pattern not being specific and the potential for the parasite to be present in lesions caused by other infectious (GD caused by *Toxoplasma* spp., *Histoplasma* spp., *Sporothrix* spp., *Blastomyces* spp., etc.) and non-infectious processes (such as sterile granuloma and pyogranuloma syndrome, granulomatous form of sebaceous adenitis), and in healthy skin due to the role played by such tissue in the epidemiology of CanL [23, 24].

Selection of the tissue samples included in the study was based exclusively on morphologic (H&E) inclusion criteria and with the authors blinded to both the presence/absence of parasite in skin lesions and to the infection status of the animals. Since a gold test result was not available, the true sensitivity and specificity of the different techniques could not be evaluated, and we therefore exclusively tested their ability to guide the histopathologic diagnosis in cases of dermal inflammation compatible with CanL cutaneous lesions.

Our results highlight that there is an increase in the detection rate of *Leishmania* when highly sensitive techniques are used, as has been described earlier [25, 26]. However, of the 23 skin samples that tested positive for *Leishmania*, 17 showed discordant results with the four techniques used, thereby confirming that there is no single suitable test for the diagnosis of cutaneous lesions caused by *Leishmania* and that histological and molecular methods need to be combined.

The best diagnostic performance was obtained by qPCR followed, in decreasing order of diagnostic performance, by cPCR/IHC and H&E staining. This finding reinforces previous findings that the qPCR method detects the presence of *Leishmania* DNA better than other techniques in both the skin and in many other tissues (i.e. blood, lymph node, spleen and bone marrow), even when conducted on FFPE tissues, which might result in DNA degradation [27, 28]. Although qPCR is an excellent tool to assess the level of exposure of a canine population to *Leishmania* infection, on the basis of the present results it does not represent the technique of choice for a histopathologist to attribute a skin lesion to *Leishmania* since its results are not associated with either the characteristics or the severity of the lesions. In addition, the feasibility of using expensive molecular techniques, such as the qPCR, in laboratories with limited resources in developing countries must be considered.

Direct microscopic examination of parasites with H&E staining is considered to be a technique specific for this purpose and used in routine evaluation. However, H&E staining has a limited sensitivity and when few amastigotes are present, these amastigotes can be easily overlooked or they can be mistaken for cellular debris phagocytosed by macrophages in an inflammatory process [29]; in both cases, the result may be an inconclusive diagnosis. IHC has been confirmed to have an improved sensitivity relative to H&E staining since it provides a higher degree of contrast between parasites and host tissues, giving unequivocal results for diagnostic purposes, even when there is a low parasitic burden. Our results are in agreement with this finding, showing that while the results for several of the analyzed skin samples (16.66%) were considered inconclusive by H&E labeling, they were confirmed in 60% of cases using IHC, with mild to moderate parasite loads. cPCR [15, 30], especially when based on kinetoplast DNA amplification, as one used in the present study, is generally considered to be more sensitive than IHC, since it amplifies a multicopy genetic target present at high copies per cell [31, 32]. However, in our study, the discriminative power of cPCR was the same as that of IHC, and the rate of agreement with qPCR was higher for IHC than for cPCR (moderate vs fair, respectively). We cannot exclude that formalin fixation could result in DNA degradation, thus reducing the power of parasite detection by this technique; however we have to consider that the amplified codon was short and the target sequence is highly repeated.

Cutaneous lesions in CanL are very pleomorphic, both from a clinical and a histologic point of view [5, 33]. They can have a different pathogenesis, with inflammatory-mediated and immune-mediated changes being the most common. Each clinical presentation has been associated with particular histologic changes involving the dermis, epidermis, hair follicles and sebaceous glands [5]. A common trait for the different clinical presentations is the presence of an inflammatory infiltrate that predominantly consists of macrophages, lymphocytes and plasma cells. Dogs with less severe clinical stages of the disease (Stage I) are more frequently characterized by nodular to diffuse patterns of inflammation, a significant higher frequency of granuloma formation and a higher detection of amastigote DNA by qPCR than dogs with more severe clinical stages (Stage II–III) [3]. Also, the frequency and intensity of granuloma formation and of a monomorphic macrophage inflammatory infiltrate have been found to be significantly associated with skin parasitism [9]. Since the more severe dermal inflammation observed in Group A samples (positive for *Leishmania* dermatitis) was more frequently characterized by a nodular and diffuse pattern, we were able to partially confirm these results. Further studies with a larger cohort and clinical information are recommended.

Due to the retrospective nature of the study, no information was available on the clinical status of the animals and the staging of the disease. However, a significant difference was demonstrated between the severity of dermal inflammatory infiltrate between Groups A and B, and the severity of dermal inflammation was associated with the parasitic skin load detected by IHC, although with a weak linear correlation. This weak link may be due to the interaction between the parasite and host being quite enigmatic; in fact, host defense against parasitic infection depends on the activation response of macrophages to the stimuli and the receptors involved and, ultimately, on the anti-inflammatory or proinflammatory profile developed [34], which makes the distribution and intensity of the inflammatory response variable.

In clinically normal skin of *Leishmania*-infected dogs that show no clinical signs and are seronegative or mildly seropositive, no histological lesions and parasite detection by IHC have been demonstrated [10]; our results partially contradict these findings because Group B included seven samples that tested negative by IHC but for which variable degrees of inflammation associated with positive cPCR or qPCR were observed. We cannot rule out that the inflammation in these cases could be related to another skin disease since (i) biomolecular results should always be interpreted cautiously [4] and (ii) inflammatory infiltrates are a common histological finding in the skin of dogs from endemic CanL areas, irrespective of the presence of parasites or evidence of infection [9].

## Conclusions

The recommendation arising from this work, and which will obviously need to be confirmed with a larger cohort of samples, is to first perform a histologic examination; once the presence of a moderate/severe LHD and/or GD has been identified, in absence of a direct demonstration of *Leishmania*, a immunohistochemical investigation should be carried out, as this test has proven to be the only technique capable of clearly defining the pathogenesis of the lesion. However, the case of mild lesions is more complex; IHC, although being able to visualize the parasite in the inflammatory cells, then loses its discriminatory power, possibly leading to an underestimation of the role of the parasite in the skin lesion. In these cases, qPCR could be of help, given its greater detection ability, although the results should be interpreted with caution and following the published guidelines for diagnosis of leishmaniosis in dogs (CLWG) [6]. In addition, in an endemic area, all history sheets of cutaneous biopsies from dogs should include the results of at least one quantitative serological test before the biopsy is sent to the pathologist.

## Data Availability

The datasets used and analyzed during the current study are available from the corresponding author on reasonable request.

## Abbreviations

CanL: Canine leishmaniosis
CLWG: Canine Leishmaniasis Working Group
cPCR: Conventional PCR
DAT: Direct agglutination test
ELISA: Enzyme-linked immunosorbent assay
FFPE: Formalin-fixed and paraffin-embedded
GD: Granulomatous dermatitis
H&E: Hematoxylin and eosin
IFAT: Immunofluorescence antibody test
IHC: Immunohistochemistry
LHD: Lymphoplasmacytic/histiocytic dermatitis
qPCR: Quantitative PCR
